# Patient preferences for conservative treatment of shoulder pain: a discrete choice experiment

**DOI:** 10.1093/fampra/cmae050

**Published:** 2024-10-10

**Authors:** Annelotte H C Versloot, Jorien Veldwijk, Ramon P G Ottenheijm, Marloes de Graaf, Daniëlle A van der Windt, Bart W Koes, Jos Runhaar, Dieuwke Schiphof

**Affiliations:** Department of General Practice, Erasmus Medical Center, Dr Molewaterplein 40, 3015 GD, Rotterdam, The Netherlands; Erasmus School of Health Policy and Management, Erasmus Choice Modelling Centre, Erasmus University Rotterdam, Burgemeester Oudlaan 50, 3062 PA, Rotterdam, The Netherlands; Department of Family Medicine, Care and Public Health Research Institute (CAPHRI), Maastricht University, P.O. Box 616 6200 MD, Maastricht, The Netherlands; Fysio-Experts, Rijndijk 137, 2394 AG Hazerswoude Rijndijk, The Netherlands; School of Medicine, Primary Care Centre Versus Arthritis, Keele University, Keele, Staffordshire, ST5 5BG, United Kingdom; Department of General Practice, Erasmus Medical Center, Dr Molewaterplein 40, 3015 GD, Rotterdam, The Netherlands; Research Unit of General Practice, Department of Public Health and Center for Muscle and Joint Health, University of Southern Denmark, Campusvej 55, DK-5230, Odense, Denmark; Department of General Practice, Erasmus Medical Center, Dr Molewaterplein 40, 3015 GD, Rotterdam, The Netherlands; Department of General Practice, Erasmus Medical Center, Dr Molewaterplein 40, 3015 GD, Rotterdam, The Netherlands

**Keywords:** patient preference, shoulder pain, primary health care, decision making, exercise therapy, conservative treatment

## Abstract

**Background:**

Shoulder pain is common amongst adults, but little is known about patients’ preferences.

**Objective:**

The aim of this study was to determine patients’ preferences for treatment options offered for shoulder pain in primary care.

**Methods:**

A discrete choice experiment was used to investigate these preferences. Adults with shoulder pain were asked to make 12 choices between two treatment options, or to opt-out. The attributes of the 12 treatment options were presented as varying in: treatment effectiveness (50%, 70%, or 90%), risk of relapse (10%, 20%, or 30%), time to pain reduction (2 or 6 weeks), prevention of relapse (yes/no), requiring injection (yes/no), and including physiotherapy (none, 6, or 12 sessions). A conditional logit model with latent class analysis was used for the analysis and a class assignment model.

**Results:**

Three hundred and twelve participants completed the questionnaire with mean age of 52 ± 15.2 years. Latent class analysis revealed three groups. Group 1 preferred to opt-out, unless the attributes were highly favorable (90% effectiveness). Group 2 preferred treatment, but not an injection. Group 3 preferred to opt-out and did not opt for treatment. The likelihood of a participant belonging to one of these groups was 68.8%, 9.3%, and 21.9%, respectively. The class assignment was related to having previously received injection or physiotherapy, as they did not prefer that same treatment again.

**Conclusion:**

This study showed that most patients with shoulder pain prefer to opt-out, unless treatment attributes are highly favorable. Characteristics of influence on this decision was whether the patient had received an injection or physiotherapy before.

**Key Messages**

- This discrete choice experiment identified potential patient groups with shoulder pain.
- Most patients with shoulder pain prefer no treatment, unless it’s highly favourable.
- Patients previously treated with an injection, did not favor that treatment again.
- Patients previously treated with physiotherapy, did not favor that treatment again.
- Future research could focus on understanding these avoidances.

## Background

Shoulder pain is common in the adult population, with an estimated incidence of 30.3 per 1000 person-years [1]. In over 50% of the patients, pain or disability last longer than 6 months [2, 3] which makes it the third most common musculoskeletal complaint seen in general practitioner (GP) practice [1, 4, 5]. The treatment of shoulder pain is often based on evidence-based treatment, the knowledge of health-care professionals, and the preferences of the patients [5].

Evidence-based conservative treatment for shoulder pain according to the Dutch GP guidelines closely aligns with the British Elbow and Shoulder Society (BESS) care pathway guideline, developed in collaboration with the National Health Services (NHS) which serve broadly as critical frameworks for clinicians [6, 7]. These guidelines initially involve patient education and prescribing analgesics [7]. If patients do not respond to this approach, subsequent recommendations include corticosteroid injections or physiotherapy, each carrying distinct advantages and disadvantages. The GP guidelines do not specify which treatment is superior. A systematic review including 177 randomized controlled trials (RCTs)  showed that a corticosteroid injection often results in short-term pain relief (< 6 weeks), but that the pain has a high chance of recurring within a year [8]. By contrast, the review also showed that physiotherapy likely results in long-term pain relief (beyond 6 weeks) and lowers the chance of recurrence within a year. The choice between these two treatments is often based on the GP’s knowledge and expertise, but also on the preferences of patients [9, 10].

Nonetheless, there is limited literature available concerning these patient preferences. A study by Smith *et al*. [11] examined the preferences for surgical treatment among patients with shoulder pain, revealing that participants were most concerned about residual pain and the duration required to return to their daily activities and work. However, there is limited evidence regarding the treatment preferences of patients with shoulder pain in primary care. Understanding these preferences could optimize care and improve therapy adherence [12], especially when multiple treatment options are considered appropriate according to guidelines. Therefore, the objective of this study was to examine patient preferences regarding conservative treatment options for shoulder pain.

## Methods

### Discrete choice experiment

A discrete choice experiment (DCE) was used to investigate patients’ preferences. DCEs are increasingly used in health care to gain a better understanding of preferences among patients and health-care providers [12]. A DCE is a quantitative method used to investigate stated preferences [13, 14]. Participants were asked to repeatedly choose between hypothetical alternatives of a health-care intervention; in this case aimed to reduce pain or disability related to shoulder pain. Each alternative in the choice tasks was described by means of different levels of characteristics (“attributes”) of the therapy, (e.g. effectiveness after 6 weeks with success rates of 50%, 70%, or 90%). When participants answer multiple choice tasks, preferences for each attribute can be determined, and relative attribute importance and subsequent secondary outcomes (such as predicted participation rates for hypothetical future treatment options) can be calculated [15]. This study was conducted using best-practice guidelines for conducting a stated preference study [16].

### Development of attributes and their levels

The selection of attributes and their corresponding levels for the DCE followed a multi-step progress. The first step was a literature search to identify potential attributes of shoulder pain treatments. Second, these identified attributes were incorporated into an interview guide. Interviews were conducted with five patients with shoulder pain (three male, two female, age ranging from 52 to 71 years), resulting in a refined list of potential attributes. In the final step, the interviewees were asked to choose three attributes which seemed most important to them. Using this information and the opinion of experts (*n* = 4, AV, JV, DS, and JR), a total of six attributes were selected. Attribute levels were selected based on input from both experts and literature to ensure they represent clinically relevant scenarios. An overview of the attributes and their levels is shown in Table 1. The explanation of the attributes and the choice tasks as presented in the questionnaire can be found in Supplementary Appendix 1.

**Table 1.** The attributes and corresponding levels for the development of a DCE on treatment preferences for patients with shoulder pain.

Attribute	Definition^*^	Levels
Effectiveness after 6 weeks	The chance that the shoulder is fully recovered after 6 weeks	50% 70% 90%
Risk of relapse after 6 months	The chance that the shoulder pain will return 6 months after treatment	10% 20% 30%
When does pain reduction occur	How long it takes for the first effect of the treatment to appear	2 weeks 6 weeks
Prevent complaints from relapse	Is there attention for the prevention of the return of shoulder pain during treatment?	Yes No
Injection	Does the treatment involve an anti-inflammatory injection?	Yes No
Physiotherapy	Does the treatment involve physiotherapy?	No Yes, 6 sessions Yes, 12 sessions

^*^Appendix 1 contains the detailed explanation provided to patients in the questionnaire.

### Experimental design of the DCE

Ngene Software was used to create a Bayesian D-efficient fractional design. A total of 12 choice tasks were included in the questionnaire. An example of a choice task is shown in Table 2. The questionnaire also included an explanation of the choice tasks, attributes and levels, and a practice question before starting the choice tasks (see Supplementary Appendix 1). Each choice task featured two alternatives for the treatment of their shoulder pain and each alternative included all six attributes with a certain level. Upon completing a choice task, participants were queried with the following question: “If you could also choose not to undergo treatment in this situation, would you prefer not to receive treatment or retain the treatment option you chose?.” This gave the participants the opportunity to opt-out (i.e. dual response design): preferring to not receive treatment above either of the treatment options. To pilot test the DCE and questionnaire, it was distributed to collect the first 10% of the data. The priors used in the pilot experimental design were based on best guesses. Based on pilot test responses, the priors in the experimental design were optimized using the estimates from the multinomial logit model.

**Table 2.** Example of a choice task included in a DCE on treatment preferences for patients with shoulder pain.

	Option 1	Option 2
Effectiveness after 6 weeks	90%	50%
Risk of relapse after 6 months	10%	30%
When does pain reduction occur	After 2 weeks	After 6 weeks
Prevent complaints from relapse	Yes	No
Injection	No	Yes
Physiotherapy	Yes, 6 sessions of 30 min	No

### Questionnaire

To prevent cognitive burden, the choice tasks were divided into two parts of six choice tasks each. Between these two parts, the questionnaire included questions about demographic information (e.g. sex, age), participants’ shoulder pain (e.g. pain duration and treatment received), health-care insurance (e.g. if they have insurance for physiotherapy), experience of treatment options (e.g. a corticosteroid injection), and comorbidities. Patient reported outcomes included the Shoulder Pain and Disability Index (SPADI) [17], Pain Coping Inventory [18], and quality of life (EQ-5D-5L [19]).

Prior to the pilot questionnaire, we selected three patients with shoulder pain (one male, two females, age ranging from 58 to 67) to test the questionnaire using a think-aloud strategy. They completed the questionnaire while reading the questions aloud and verbally articulating their thought processes, resulting primarily in textual alterations.

### Study sample and recruitment

The sample size calculation was based on standard sample size calculations used for a DCE and resulted in a minimum sample size of around 300 participants [20, 21]. Participants were recruited through Dynata, a commercial survey sample provider. Participants could participate if they gave informed consent and met the following criteria: (i) age 18 years or older, (ii) currently having shoulder pain or having experienced shoulder pain in the past 2 years, (iii) no traumatic origin of the shoulder pain. All participants who completed the questionnaire received a financial compensation of €6.50.

### Statistical methods

Participant characteristics were analyzed using IBM SPSS Statistics for Windows, version 28 (IBM Corp., Armonk, N.Y., USA). Descriptive statistics for numerical variables involved mean and standard deviation, while for categorical variables frequency counts and percentages were used.

DCE analyses were conducted in Nlogit software and results were considered significant when *P* < .05. Panel Latent Class Models (LCM) were used to determine the preferences, as such models take into account the multilevel structure of the data (i.e. every participant answered 12 choice tasks) [15, 22]. By using a latent class model it can be determined whether different preferences exist across unobserved subgroups of the population. In LCA participants are not allocated to only one specific class; each participant has a certain probability to belong to a class. In order to determine the number of classes, we selected the model with the best fit based on the Akaike Information Criterion, Bayesian Information Criterion, and loglikelihood criteria (models ranging from one to five classes were tested). Demographic measures can be incorporated into the LCM modeling procedure, which provides some insight into which participants are more or less likely to belong to what class.

All attributes were considered non-linear and recoded using effect codes [23]. The final utility equation used in the LCM was:


$$\begin{array}{l} \begin{array}{lllllll} & V_{rta|c}=\beta 1_{|c}\;Ef\;fectivenes{s_{70\%}}_{\;rta|c}+\beta 2_{|c}\;Ef\;fectiveness_{90\%}{\;}_{rta|c}\; & +\beta 3_{|c}\;Relapse\;risk_{20\%}{\;}_{rta|c}+\beta 4_{|c}\;Relapse\;risk_{30}{\;}_{rta|c}\; & +\beta 5_{|c}\;Pain\;reduction_{6}{\;}_{weeks\;rta|c}+\beta 6_{|c}\;Prevention\;of\;relapse_{Yes\;rta|c}\; & +\beta 7_{|c}\;Injection_{Yes\;rta|c}+\beta 8_{|c}\;Physiotheraph{y_{6}}_{sessions\;rta|c}\; & +\beta 9_{|c}\;Physiotherapy_{12\;}{\;}_{sessions\;rta|c}\; & V_{opt-out}=\beta 0_{|c} \end{array} \end{array}$$

The systematic utility component (V) describes the observable utility that participant “r” belonging to class “c” reported for alternative “a” in choice task “t.” The β_0_ represents the alternative specific constant for no treatment, while β_1_ – β_9_ represent the attribute level estimates.

A class assignment model was fitted to assess the contribution of participant characteristics (i.e. age, gender, education level, previous injection, previous physiotherapy, insurance package, currently having shoulder pain or not). A significant variable in the class assignment model indicates that this variable contributes to the class assignment (e.g. if the coefficient of the age variable is positive and significant for class 1, participants with a higher age are more likely to belong to class 1).

Relative importance scores for the attributes relative to the most important attribute were calculated based on the results of the LCMs, separately for each class. The class adjusted relative importance was calculated by computing the relative importance score of all attributes in each class separately after which they were weighted according to class assignment probability.

We further calculated the predicted participation rates (estimated percentage of individuals who would choose that option) for three potential treatment options. Each hypothetical treatment selection scenario consisted of two potential treatment options and an opt-out option. The first scenario includes a “quick-fix” injection treatment option and a treatment option with physiotherapy which shows better results in the long run. The second scenario investigates what participants will choose if the effectiveness of the two treatment options is the same after 6 weeks, but differs in the other attributes. One treatment option includes an injection with quick pain relief but no attention to prevention and a high risk of relapse and the other treatment option includes physiotherapy sessions and attention to prevention with a low risk of relapse. The third scenario has a treatment option with only a corticosteroid injection and a treatment option with both a corticosteroid injection and physiotherapy resulting in better outcomes.

Predicted choice 1/(1 + exp^-v^) between different hypothetical treatment options compared to no treatment were tabulated using LCM model estimates for each class.

## Results

### Participants characteristics

A total of 312 participants gave informed consent and filled in the questionnaire. Of these participants, 209 currently reported shoulder pain (67%), the remainder had experienced shoulder pain in the previous 2 years. The mean age of the whole group was 52 years (standard deviation (sd) = 15.2) and 45.8% were men. The majority of the group, 59.6%, was employed and 43.4% had an intermediate or higher educational level. The shoulder pain had started spontaneously in 40.1% and most participants reached out to a health-care professional: 47.8% went to a GP and 52.6% to a physiotherapist.

Between the group with current shoulder pain and the group with previous shoulder pain, there were no major differences for most baseline characteristics. Only the duration of pain showed a difference between the two groups. The participants with current shoulder pain showed a mean duration of 145.8 weeks (sd = 793.8), whereas the participants with previous shoulder pain reported a mean duration of 7 weeks at that time (sd = 12.5). This group also consulted the GP and the physiotherapist less often (Table 3).

**Table 3.** Patient characteristics of 312 patients with current shoulder pain or shoulder pain in the past 2 years participating in a discrete choice experiment on treatment preferences for patients with shoulder pain (2023).

Variable	Current shoulder pain (*n* = 209)	Shoulder pain in past 2 years (*n* = 103)	Total (*n* = 312)
Age, mean ± sd	52.4 ± 15.8	51.2 ± 14.1	52.0 ± 15.2
Sex, *N* (%) Man Woman Non-binary	92 (44) 116 (56) 1 (1)	51 (50) 52 (51) 0	143 (46) 168 (54) 1 (0)
BMI, mean ± sd	26.8 ± 6.3	26.3 ± 6.2	26.6 ± 6.3
Education level, *N* (%) Low Intermediate High	45 (22) 86 (41) 78 (37)	15 (15) 49 (48) 39 (38)	60 (19) 135 (43) 117 (38)
Dutch Nationality, *N* (%)	201, (96)	96 (93)	297 (95)
Employment status, *N* (%) School Employed Unemployed Retired	3 (1) 115 (55) 44 (21) 47 (23%)	2 (2) 68 (66) 14 (14) 19 (18)	5 (2) 183 (59) 57 (18) 67 (22)
SPADI scores, mean ± sd	46.0 ± 22.4	35.7 ± 25.6	42.6 ± 23.9
Duration of the pain in weeks, mean ± sd	145.8 ± 793.8	7.0 ± 12.5	–
Presumed cause of shoulder pain, *N* (%) Spontaneous Due to hobby or work Sports Other	83 (40) 61 (29) 14 (7) 51 (24)	42 (41) 7 (7) 37 (36) 17 (17)	125 (40) 68 (22) 51 (16) 68 (22)
Shoulder pain in history, *N* (%)	131 (63)	–	–
Trauma or surgery in history^*^, *N* (%) Fracture Tendon rupture Dislocated Surgery Other	30 (14) 3 (1) 8 (4) 4 (2) 9 (4) 7 (3)	9 (9) 2 (2) 2 (2) 3 (3) 3 (3) 0	39 (13) 5 (2) 10 (3) 7 (2) 12 (4) 7 (2)
GP consultation, *N* (%) Treatment*: Education/advice Painkillers Referral to physiotherapy Injection Referral to a hospital specialist Referral for a X-ray or MRI Other	111 (53) 19 (9) 53 (25) 69 (33) 23 (11) 22 (11) 21 (10) 9 (4)	38 (37) 9 (9) 19 (18) 22 (21) 9 (9) 2 (2) 2 (2) 1 (1)	149 (48) 28 (9) 72 (23) 91 (29) 32 (10) 24 (8) 23 (7) 10 (3)
Physiotherapy consultation, *N* (%) Treatment^*^: Massage Joint mobilizations Physiotherapy Other	123 (59) 86 (41) 23 (11) 80 (38) 6 (3)	41 (40) 25 (24) 8 (8) 23 (22) 2 (2)	164 (53) 111 (36) 31 (10) 103 (33) 8 (3)

BMI = body mass index, SD = standard deviation, GP = general practitioner.

^*^Multiple responses per participant were permitted.

### Preferences regarding shoulder treatment characteristics

The latent class analysis was used to investigate preferences of the population for treatment characteristics as well as differences in preferences within this population. The analysis showed three latent classes which are presented in Table 4. If the coefficient is significant, it means that the attribute is of significant importance in the decision making process of the participants in this class. A positive coefficient refers to attributes contributing to the total positive value of a treatment while a negative coefficient shows a disutility. For example, in class 1 the attribute “effectiveness” has significant levels for 50% and 90% (estimate = -0.69 vs 0.71), with, 90% effectiveness preferred over 50%.

**Table 4.** Attribute level estimates, model fit, and class assignment results of the latent class analysis of 312 patients with current shoulder pain or shoulder pain in the past 2 years participating in a discrete choice experiment on treatment preferences for patients with shoulder pain (2023).

		Class 1		Class 2		Class 3	
		Coefficient	SE	Coefficient	SE	Coefficient	SE
Effectiveness	50% (ref)	−0.69***	–	−0.47***	–	0.00	–
	70%	−0.01	0.05	0.03	0.07	0.01	0.14
	90%	0.71***	0.05	0.44***	0.09	0.00	0.16
Risk of relapse	10% (ref)	0.24***	–	0.08	–	-0.20	–
	20%	0.07*	0.04	−0.01	0.07	0.22	0.15
	30%	−0.31***	0.05	−0.06	0.08	−0.02	0.14
Pain reduction	2 weeks (ref)	0.17***	–	0.13***	–	0.11	–
	6 weeks	−0.17***	0.03	−0.13***	0.05	−0.11	0.10
Prevention of relapse	No (ref)	−0.29***	-	0.02	–	−0.17*	–
	Yes	0.29***	0.03	-0.02	0.05	0.17*	0.11
Injection	No (ref)	−0.10***	–	0.58***	–	0.13	–
	Yes	0.10***	0.03	-0.58***	0.09	−0.13	0.11
Physiotherapy	None (ref)	−0.10	–	0.09	–	0.67***	–
	6 sessions	0.08*	0.04	0.10	0.07	0.02	0.16
	12 sessions	0.02	0.05	-0.18*	0.10	−0.69***	0.22
Opt-out		2.07***	0.20	-0.36***	0.11	2.06***	0.18
Model fit							
Restricted log lokelihood		−4100.02106					
AIC		6517.4					
Class assignment model							
Constant		3,32***	0.70	1.02	0.98	–	–
Previous physiotherapy		−1,50***	0.51	−1.86***	0.61	–	–
Previous injection		−0.10	0.48	1.12**	0.57	–	–

Ref = reference category, SE = standard error.

*≤0.05 significance, **≤0.01 significance, ***<0.001 significance.

For classes 1 and 3, participants preferred the opt-out over treatment (positive coefficients for opt-out) and in class 2 participants preferred treatment over opt-out (negative coefficient for opt-out).

Each participant has a probability to belong to one of these classes based on their preferences.

Class 1 has a 68.8% probability of participants belonging to this class and will be called the “optimal effectiveness seekers.” All attributes had one or more significant levels for this class, so all attributes were important. They preferred 90% effectiveness over 50% and a 10% risk of relapse over 30%. They preferred pain relief after 2 weeks over 6 weeks and preferred if there was attention for prevention over no attention. They preferred a corticosteroid injection and six sessions of physiotherapy over none or 12 sessions.

Class 2 has a 9.3% probability of participants belonging to this class and will be called the “non-injection seekers.” The following attributes had one or more significant levels: participants preferred 90% effectiveness over 50% and pain relief after 2 weeks over 6 weeks. They did not prefer a corticosteroid injection and they did not prefer 12 sessions of physiotherapy.

Class 3 has a 21.9% probability of participants belonging to this class and will be called the non-treatment seekers’. There were only two attributes significant. Participants preferred if there was attention to prevention during treatment and they preferred no physiotherapy over 6 or 12 sessions.

**Figure 1.**
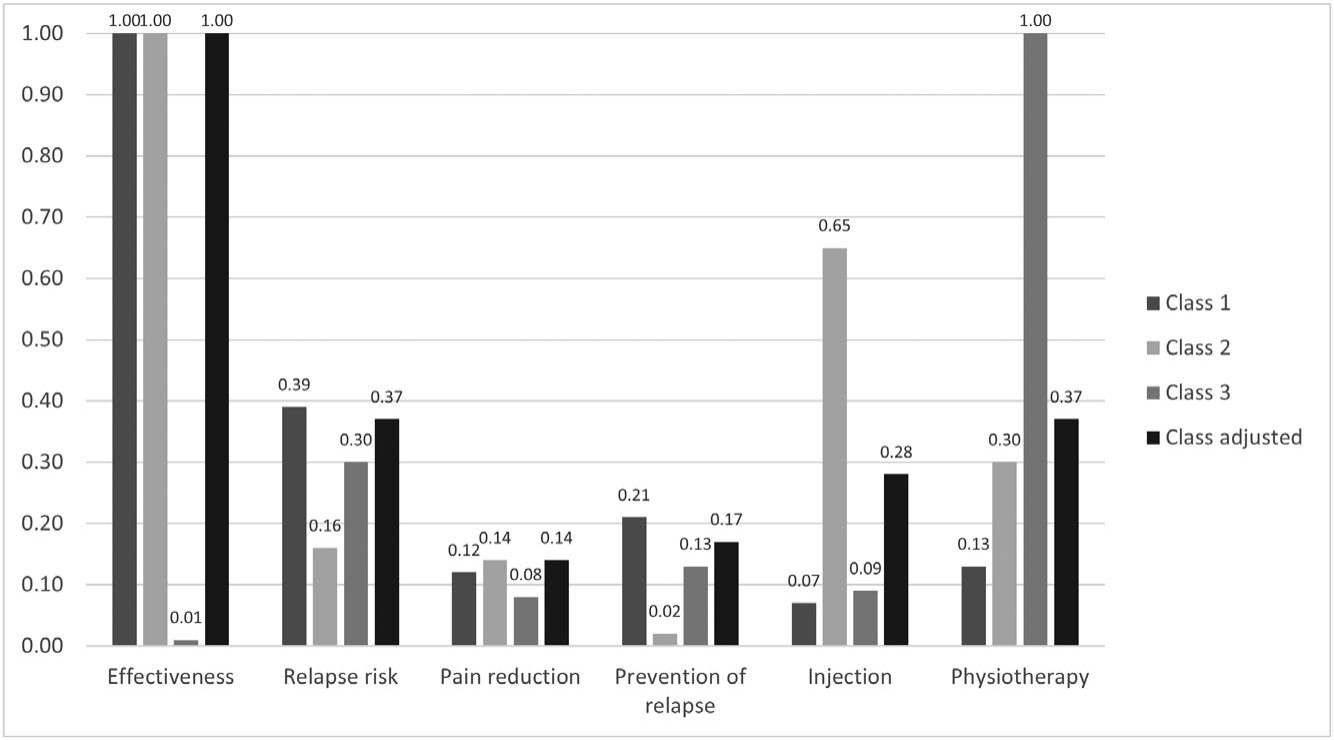
Relative importance of attributes separately for all classes of the latent class model as well as adjusted for class probability based on a discrete choice experiment on treatment preferences for patients with shoulder pain including 312 patients with current shoulder pain or shoulder pain in the past 2 years (2023).

The distinct differences in preferences across the classes is also shown in Fig. 1, displaying the relative importance of the attributes for each class and the class adjusted probability. This probability accounts for the likelihood that an individual belongs to multiple classes and combines the preferences of all classes.

### Causes of differences in preferences across the patient population

The class assignment model was used to assess the contribution of participant characteristics. The likelihood to belong to one of the three classes depended only on whether participants received an injection or physiotherapy in the past, as seen in Table 4. The other characteristics were not significantly associated with class assignment. Participants who had previously received physiotherapy were more likely to belong to class 3 (the non-treatment seekers) compared to class 1 and 2. Participants who had an injection before were more likely to belong to class 2 (the non-injection seekers) compared to class 3.

### Uptake of hypothetical scenarios

The predicted uptake of three hypothetical scenarios is presented in Table 5. Across all calculations, on average, over half of the participants would opt-out, however class-specific uptake rates were highly variable. Especially class 1, the optimal effective seekers, and class 3, the non-treatment seekers, often chose to opt-out.

**Table 5.** Predicted uptake of hypothetical scenarios of a discrete choice experiment on treatment preferences for patients with shoulder pain including 312 patients with current shoulder pain or shoulder pain in the past 2 years (2023).

Scenario 1	Option 1	Option 2	Opt-out
Effectiveness	90%	50%	–
Risk of relapse	30%	10%	–
Pain reduction	2 weeks	6 weeks	–
Prevention of relapse	No	Yes	–
Injection	Yes	No	–
Physiotherapy	No	12 sessions	–
Uptake class 1	13%	7%	80%
Uptake class 2	40%	33%	27%
Uptake class 3	16%	5%	79%
Class adjusted uptake	21%	14%	65%

Scenario 2	Option 1	Option 2	Opt-out
Effectiveness	70%	70%	-
Risk of relapse	30%	10%	-
Pain reduction	2 weeks	6 weeks	-
Prevention of relapse	No	Yes	-
Injection	Yes	No	-
Physiotherapy	No	6 sessions	-
Uptake class 1	6%	14%	80%
Uptake class 2	21%	58%	21%
Uptake class 3	15%	10%	75%
Class adjusted uptake	12%	26%	62%

Scenario 3	Option 1	Option 2	Opt-out
Effectiveness	70%	90%	–
Risk of relapse	30%	10%	–
Pain reduction	2 weeks	2 weeks	–
Prevention of relapse	No	Yes	–
Injection	Yes	Yes	–
Physiotherapy	No	6 sessions	–
Uptake class 1	5%	36%	59%
Uptake class 2	27%	45%	28%
Uptake class 3	15%	9%	75%
Class adjusted uptake	13%	35%	52%

Scenario 1 showed that if participants choose a treatment, they will prefer treatment option 1, the “quick fix” injection, compared to option 2, physiotherapy with better results in the long run.

Scenario 2 showed that if participants choose between two treatments with the same effectiveness, they will prefer treatment option 2. This option included physiotherapy with attention to prevention and a low risk of relapse.

Scenario 3 showed that if participants choose a treatment, they will prefer treatment option 2 including both an injection and physiotherapy associated with a better effectiveness and a low risk of relapse.

The analysis also showed that class 2, the non-injection seekers, preferred the option with physiotherapy, unless the corticosteroid injection was 40% more effective then physiotherapy. In comparison, it showed that class 3 would not choose physiotherapy even if that option is more effective and has a lower risk of relapse than a corticosteroid injection.

## Discussion

This study is the first to investigate patient preferences regarding conservative treatments for shoulder pain in primary care. It was found that participants tended to avoid treatment, unless the treatment offered favorable characteristics, such as a high effectiveness or a reduced change of relapse. However, there will also be a small group who will be hard to convince to have treatment. There is also reluctance among patients previously treated with physiotherapy or a corticosteroid injection to opt for such treatment again.

It was remarkable that the participants tended to avoid treatment, even though they experienced shoulder pain. Multiple qualitative studies investigated treatment experiences of patients with shoulder pain. Two studies found that patients waited a long time before contacting a health-care professional, since they expected that the pain would resolve spontaneously [24, 25]. Only if the pain was severe enough, such as keeping them awake at night or not being able to work anymore, they would visit a health-care professional. The SPADI scores in table 1 indicate a mean score of 42.6 which corresponds with moderate shoulder pain or disability. This could explain why many participants in this study did not prefer receiving treatment, since their problem, in their eyes, was not severe. Another possible explanation is that the mean duration of pain for the group experiencing current shoulder pain was three years. These patients probably had undergone various treatments with disappointing outcomes, potentially leading to their reluctance to pursue further treatment options. However, it is known that starting early with treatment, such as physiotherapy, results in better outcomes compared to a wait-and-see policy [26], so it is recommended to communicate this to patients.

Another remarkable finding is that patients who previously had physiotherapy or received an injection were hesitant to pursue the same treatment for their shoulder pain. This only changed if the treatment had promising attributes, such as a high effectiveness or a low risk of relapse. This may suggest that previous experiences with physiotherapy or an injection might not have been positive. This was also observed in a qualitative study of Subialka *et al*. [27]. This study showed that patients undergoing physiotherapy for musculoskeletal conditions often experienced that it was a longer trajectory than expected [27]. Participants in this study also experienced receiving varying recommendations from different health-care professionals regarding continuing with physiotherapy or trying other treatment options, such as a corticosteroid injection, which led to confusion. In general, they experienced a lack of information provision. Other studies regarding primary care management for shoulder pain found that patients were dissatisfied with the limited assessment and education provided by GPs [24, 28]. This is also seen in a study investigating the experiences of patients receiving an intra-articular injection. They found that patients wished to receive more information prior to the intervention and that only 50% of the patients experienced much improvement after the injection [29]. This might explain why patients are hesitant receiving an injection again. Another explanation may be that the average duration of symptoms in the group of this study currently experiencing shoulder pain was approximately 3 years. This indicates that this group also consisted of patients with a chronic condition, known to contribute to therapy resistance [30].

### Limitations

A limitation of this study is its contextuality as the findings are specific to the health-care setting in the Netherlands. That makes the findings representative for the Dutch health-care system, but the generalizability may be limited to other countries due to a variation in health-care systems, health-care insurances, and guidelines for the treatment of shoulder pain. However, the Dutch guideline for shoulder pain is similar to the BESS care pathway guideline which is made in collaboration with the NHS and are used worldwide. Therefore, this research could serve as a valuable framework for conducting similar research in other countries.

Another limitation of this study is the risk of selection bias, since the participants were not randomly selected to complete the questionnaire. The participants were also not recruited at the time they sought help for their shoulder pain, particularly those who had previously experienced shoulder pain. This could lead to potential recall bias. However, the data showed heterogeneity and was a good representation of the population with shoulder pain compared to a cohort study that prospectively included individuals with shoulder pain in primary care [1].

A strength of this research is the heterogeneity within the sample which made it possible to use the latent class analysis. Another strength is the design of the DCE and the questionnaire. The DCE was designed first using a multi-step strategy involving a literature search, interviews with the target population and expert opinions. Secondly, the questionnaire was tested using the think-aloud strategy with patients of the target group. Finally, the incorporation of a pilot phase, to test the questionnaire after the first 10% of the data was collected and analyzed, resulted in an optimized DCE design.

Overall, the findings of this study emphasize the need to better inform patients about treatment options and to investigate barriers to accessing recommended treatment options. For example, information about the importance of symptom duration, the effectiveness of physiotherapy and injections, and information about injections to diminish fear, could help make a better choice. It is also necessary to gather insights into the barriers participants encountered that led to their hesitancy in opting for treatment, as well as the reasons they declined previously received treatments. By closely examining these barriers, health-care professionals can adapt their educational strategies to more effectively address the needs and concerns of patients and to dispel misinformation. This will result in improved alignment in patient expectation management.

## Conclusions

The results of this DCE showed that patients with shoulder pain often prefer to opt-out, unless the other attributes are favorable, such as a high effectiveness (90% success rate). Patients who previously received physiotherapy or an injection did often not favor that same treatment for their shoulder pain. This DCE identified the potential patient groups with shoulder pain a GP may encounter in practice and highlighted the necessity of providing comprehensive information about treatments to improve patient expectation management.

## Supplementary Material

cmae050_suppl_Supplementary_Appendix_1

## Data Availability

The data underlying this article will be shared on reasonable request to the corresponding author.
