# Supplementary material for: Patient preferences for conservative treatment of shoulder pain: a discrete choice experiment
Source: Fam Pract. 2024 Oct 10;42(2):cmae050. doi: 10.1093/fampra/cmae050 (PMC11878378; doi:10.1093/fampra/cmae050)
Supplement: cmae050_suppl_Supplementary_Appendix_1 [file cmae050_suppl_supplementary_appendix_1.pdf]

APPENDIX 1: Explanation of the choice tasks in the questionnaire

Explanation of choice tasks

In the next part of the questionnaire, we present you twice with 6 choice tasks about the treatment of shoulder pain. We ask you to choose the option you would prefer for the treatment of shoulder complaints. If you currently have no symptoms, make the choice by moving back in time when you did have shoulder symptoms. Each choice task includes 2 options that can sometimes be similar, yet different. That is why we ask you to read the explanation below carefully. Because this is about your opinion, there are no right or wrong answers.

Below is first an explanation of some of the characteristics of the treatment for shoulder pain. It is important that you read this carefully first. This is followed by an example.

Effectiveness after 6 weeks:

This is the probability that the shoulder has fully recovered, after 6 weeks. This chance is expressed in percent, for example 70%. That is, out of every 100 patients, 70 will be fully recovered after 6 weeks and 30 will not be fully recovered after 6 weeks.

Chance of recurrence of symptoms after 6 months:

This is the probability that symptoms will return 6 months after treatment. This probability is expressed as a percentage, for example, 40%. That is, out of every 100 patients, there will be 40 where the symptoms returned within 6 months and 60 where the symptoms did not return within 6 months.

When does pain reduction occur:

This is how long it takes before you notice the first effect of the treatment on your pain symptoms. This is expressed in weeks, for example: pain decreases after 2 weeks.

Prevention of recurrence of symptoms:

This indicates whether the treatment focuses on preventing the shoulder pain from coming back.

Injection with an anti-inflammatory drug:

Shoulder pain can be treated by your doctor through a corticosteroid injection. This is an anti-inflammatory drug. The injection is given once in or around the shoulder joint.

Exercise therapy under the supervision of a physical therapist:

Shoulder problems can be treated by a physical therapist through exercise therapy, in which the mobility of the shoulder is improved and recovery is promoted.

Example of a choice task

The following is an example (you do not need to complete this question):

Imagine that for your shoulder pain you get to choose which treatment you get, which of the following options would you prefer, option 1 or option 2?

	**Option 1**	**Option 2**
**Effectiveness after 6 weeks**	90%	50%
**Risk of relapse after 6 months**	10%	30%
**When does pain reduction occur**	After 2 weeks	After 6 weeks
**Prevent complaints from relapse**	Yes	No
**Injection**	No	Yes
**Physiotherapy**	Yes, 6 sessions of 30 minutes	No

I will choose

- ● Option 1
- ○ Option 2

In this example, option 1 was chosen. This choice gives a 90% chance that the shoulder will be fully recovered after 6 weeks. The chance of the symptoms returning after 6 months is 10%. Pain reduction occurs after 2 weeks. During treatment, there will be attention to preventing the recurrence of symptoms. There is no injection and 6 sessions of 30 minutes of exercise therapy take place as treatment.
After this you may make your own choices. In total there are twice 6 choice tasks. First consider carefully which of the above characteristics you consider important in the treatment. Now follow the first 6 choice tasks.
